# Entropy-Stabilized Oxides owning Fluorite Structure obtained by Hydrothermal Treatment

**DOI:** 10.3390/ma13030558

**Published:** 2020-01-24

**Authors:** Luca Spiridigliozzi, Claudio Ferone, Raffaele Cioffi, Grazia Accardo, Domenico Frattini, Gianfranco Dell’Agli

**Affiliations:** 1 Department of Civil and Mechanical Engineering, University of Cassino and Southern Lazio, Via G. Di Biasio 43, 03043 Cassino (FR), Italy; l.spiridigliozzi@unicas.it; 2 Department of Engineering, Università di Napoli “Parthenope”, Centro Direzionale, Isola C4, 80143 Napoli (NA), Italy; claudio.ferone@uniparthenope.it (C.F.); raffaele.cioffi@uniparthenope.it (R.C.); 3 INSTM - National Interuniversity Consortium of Materials Science and Technology, Via G. Giusti 9, 50121 Florence, Italy; 4 Center of Hydrogen-Fuel Cell Research, Korea Institute of Science and Technology, Hwarangno 14-gil, Seongbuk-gu, Seoul 136-791, Korea; d16605@kist.re.kr; 5 Graduate School of Energy and Environment, Seoul National University of Science and Technology, 232 Gongneung-ro, Nowon-gu, Seoul 01811, Korea; domenico.frattini@seoultech.ac.kr

**Keywords:** entropy-stabilized oxides, hydrothermal treatment, fluorite structure

## Abstract

Entropy-Stabilized Oxides (ESO) is a modern class of multicomponent advanced ceramic materials with attractive functional properties. Through a five-component oxide formulation, the configurational entropy is used to drive the phase stabilization over a reversible solid-state transformation from a multiphase to a single-phase state. In this paper, a new transition metal/rare earth entropy-stabilized oxide, with composition Ce_0.2_Zr_0.2_Y_0.2_Gd_0.2_La_0.2_O_2−δ_, was found after several investigations on alternative candidate systems. X-Ray Diffraction (XRD) analyses of calcined powders pointed out different behavior as a function of the composition and a single-phase fluorite structure was obtained after a specific thermal treatment at 1500 °C. Powders presented the absence of agglomeration, so that the sintered specimen exhibited sufficient densification with a small porosity, uniformly distributed in the sample.

## 1. Introduction

Since ancient history, materials science strongly influenced our society, and the discovery of new classes of materials creates exciting research opportunities and possible novel technological breakthroughs. In fact, the identification and engineering of new materials, with improved properties, lower production costs, lower environmental impact, and higher environmental compatibility are crucial for further developments of our technology.

Very recently (i.e., 2015), aiming to extend the study of the configurational entropy effect upon High Entropy Alloys (HEAs) [1] over non-metallic systems, Rost et al. [2] demonstrated the existence of the so-called Entropy Stabilized Oxides (ESOs) by successfully synthesizing a single-phase 5-component oxide, (Mg_0.2_Co_0.2_Ni_0.2_Zn_0.2_Cu_0.2_)O, with a rock salt structure stabilized by the configurational entropy. In particular, to be “entropy-stabilized”, a system must have both high configurational entropy and positive forming heat (+ΔH), thus being the entropy that controls its thermodynamic stability. Additionally, the reversibility between low-temperature multiphase and high-temperature single phase is a mandatory requirement of entropy-driven stabilization. In fact, even if several researchers interchangeably use the terms High Entropy Oxides (HEOs) and Entropy Stabilized Oxides (ESOs), they refer to two different concepts [3]. The former strictly refers to multicomponent systems with high configurational entropy, while the latter refers correctly to Rost’s definition of “entropy-stabilization”. In many high-entropy materials recently reported in the literature, the demonstration of phase reversibility is missing [4,5,6].

Starting from the pioneering work of Rost et al., many efforts have been spent on the synthesis of novel multicomponent ESO systems with the rock salt structure, focusing on their surprising properties [7,8,9], sinterability [10], etc. However, the theoretical concept of ESOs can be extended to other crystal structures [3], as well as to rare earth and transition metal oxides, thus an understanding of the full potential of ESOs (both rock-salt-structured or with different crystallographic structures) is still far from being unlocked.

The importance of synthesizing rare earth ESOs is mainly driven by the technologically interesting properties of rare earth oxides [11,12,13,14], especially when doped with other cations (either aliovalent or isovalent). These doped oxides are commonly used for a wide range of applications, such as sunscreen cosmetics, magneto-optical devices, catalysts, biomarkers, colorant for special glasses, solid electrolytes, etc. [15,16,17].

Djenadic et al. suggested three “selection rules” as a guide to choose the equiatomic compositions prone to producing single-phase HEOs: cations should have similar ionic radii, at least one of the possible binary oxide systems should have a different crystal structure, and at least one binary oxide pair should not have complete miscibility on the oxide binary phase diagram [4]. Furthermore, Sarkar et al. demonstrated that the presence of Ce^4+^ is crucial to stabilize the desired single-phase structure in multicomponent rare earth systems; therefore, it is reasonable to consider CeO_2_ as the parent structure in such HEO systems [5].

Therefore, in light of these considerations and the results of Chen et al. [3], we synthesized several systems in which 40% of the selected cations form fluorite-structured oxides. In particular, the cations selected in the present works are Ce and Zr as cations forming fluorite-structured oxides and the trivalent cations (with fixed valence) Y, Yb, La, Gd and Er as cations forming non-fluorite-structured oxides.

All the tested 5-component equiatomic systems have been synthesized via hydrothermal treatment at low temperature (120 °C), aiming to obtain non-agglomerated and easily sinterable powders. The benefits of this synthesis method on powders’ quality is well known [18,19], even in the case of ESOs [10]. 

The results of the present study demonstrate that the (Ce_0.2_Zr_0.2_Y_0.2_La_0.2_Gd_0.2_)O_1.7_ sample can be considered an ESO, and its entropy-driven single-phase stabilization has been tested through a heat treatment at different temperatures (ranging from 750 to 1500 °C) and air quenching to room temperature. After calcination and air quenching at 1100 °C, phase segregation has been observed, while after calcination of the same sample at 1500 °C (air-quenched from 1200 °C), the single-phase fluorite-like structure has been reversibly restored. To the best of our knowledge, it is the first time that an ESO-exhibiting fluorite crystal structure has been obtained in the presence of only 40% of cations giving rise to that crystal structure. 

Definitely, the synthesized (Ce_0.2_Zr_0.2_Y_0.2_La_0.2_Gd_0.2_)O_1.7_ sample can be reversibly transformed from a low-temperature multiphase to a high-temperature single-phase, thus demonstrating that it is a new fluorite-structured ESO. Differently from the full conversion to single-phase rock-salt-structured systems occurring between 850 and 900 °C [2,10], the full conversion to a single-phase fluorite-structured system reported here occurs between 1100 and 1200 °C, thus possibly suggesting that fluorite-structured ESOs have higher positive formation enthalpies than the rock-salt-structured ones.

## 2. Materials and Methods

Ce(NH_4_)_2_(NO_3_)_6_ and ZrO(NO_3_)_2_ (both from Carlo Erba, Milan, Italy), and Y(NO_3_)_3_∙6H_2_O, Gd(NO_3_)_3_∙6H_2_O, La(NO_3_)_3_∙6H_2_O, Yb(NO_3_)_3_∙5H_2_O, Er(NO_3_)_3_∙5H_2_O (all from Sigma Aldrich, Milan, Italy) were used as metal precursors, while an ammonia solution 30 wt% (Carlo Erba, Milan, Italy) and urea, CO(NH_2_)_2_, (purity > 99%, Carlo Erba, Milan, Italy) were used as precipitating/mineralizing agents for the hydrothermal syntheses. The choice of ammonia and urea as precipitating/mineralizing agents depended on the following considerations: on one hand, we intended to use basic precipitating/mineralizing agents for the hydrothermal treatments in order to avoid possible metals contamination in the as-synthesized phases (so we excluded alkaline hydroxides) and on the other hand, in some of our previous works, we already used both urea and ammonia for the synthesis of doped/co-doped ceria-based compounds, obtaining very different precursors in terms of morphology and obtained phases [20,21,22]. All the chemicals were used as received without any further purification.

Regardless of the actual composition, which always constituted 5 different cations, in all the samples, the molar fraction of each cation was 0.2, i.e., the generic chemical formula was (A_0.2_B_0.2_C_0.2_D_0.2_E_0.2_)O_1.7_, where A and B were always Ce and Zr, respectively, and C, D, and E were selected among Y, Gd, Yb, La, Er. 

The procedure for the hydrothermal syntheses in the presence of NH_3_ can be summarized as follows:

1. All nitrates were dissolved in demineralized water with total cation concentration equal to 0.1 M (so the molarity of each cation was 0.02 M). This solution (solution A) was vigorously stirred to favor the dissolution.
2. The mineralizer solution was formed by using a volume of NH_3_ solution (solution B) equal to 1/5 of the volume of solution A. The ammonia concentration of solution B is 30 wt%.
3. Solution B was quickly added to the selected volume of solution A and maintained under mild stirring. When the solution B was added to solution A, a precipitate instantly formed.
4. The as-prepared suspensions were immediately transferred in Teflon vessels (60 mL), which were then sealed and held in outer stainless-steel pressure vessels for the hydrothermal treatment. The treatment was carried out in an air-thermostated (at 120 °C) oven with the vessels rotating at 25 rpm to allow a very high mixing uniformity in the system all over the process duration. The duration of the treatment was fixed to 60 h.
5. Eventually, the vessels were quenched with cold water and the resulting products were repeatedly filtered by using a vacuum pump, washed with deionized water, and finally dried overnight at 80 °C under static air.

In the case of urea as a precipitating/mineralizing agent, the steps b) and c) were not present and a appropriate amount of urea (in order to have the molar ratio urea/total cations = 20) was directly added into solution A, which was directly transferred into a Teflon vessel. When the solution reached a temperature higher than 85 °C during the hydrothermal treatment, urea decomposed, giving rise to an alkalization and the eventual formation of a precipitate.

The various synthesized samples are identified with a label reporting all the cations present and the mineralizer used. For example, CeZrYLaGd-N and CeZrYLaGd-U indicate samples obtained using Ce, Zr, Y, La and Gd as cations, and synthesized using NH_3_ (N) or urea (U) as the precipitating/mineralizing agent, respectively. 

The as-synthesized powders underwent several calcination steps (at temperatures in the range 750–1500 °C) in order to reveal the possible formation of entropy-stabilized oxides. The best powders were uniaxially pressed at 1100 tons for 120 s to form pellets and finally fired at 1500 °C for 4 h to reveal the sintering behavior.

All samples were characterized by X-ray powder diffraction (XRD) using a diffractometer Miniflex 600 (Rigaku, Tokyo, Japan) and CuK radiation to detect the crystalline phases. The lattice parameters for the various samples were extracted by least square refinement.

The thermal behavior of some samples was investigated through a STAR analyzer (Mettler-Toledo, Columbus, OH, USA) in air; with a heating rate of 10 °C/min up to 1200 °C and using α-Al_2_O_3_ as a reference.

Powders’ morphology and sintered pellets’ microstructure were studied by Scanning Electron Microscopy, SEM, (Inspect F, Fei Co., Hillsboro, OR, USA) equipped with the Energy-Dispersive X-ray Spectroscopy (EDS) apparatus.

## 3. Results and Discussion

**Figure 1:**
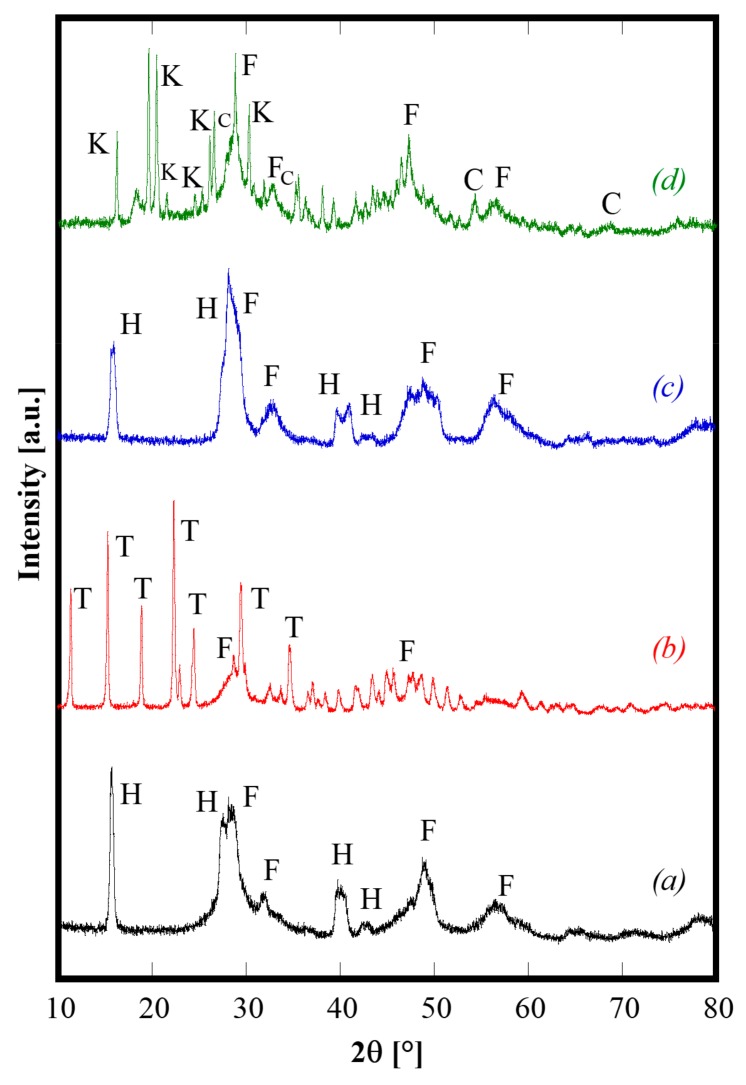
Diffraction patterns of CeZrYLaGd-N (pattern *a*), CeZrYLaGd-U (pattern *b*), CeZrYbLaGd-N (pattern *c*) and CeZrYbLaGd-U (pattern *d*) samples hydrothermally synthesized at 120 °C for 60 h. H, F, T, K and C label Hydroxides, Fluorite, Tengerite, Kozoite and Oxy-carbonate phases, respectively.

**Figure 2:**
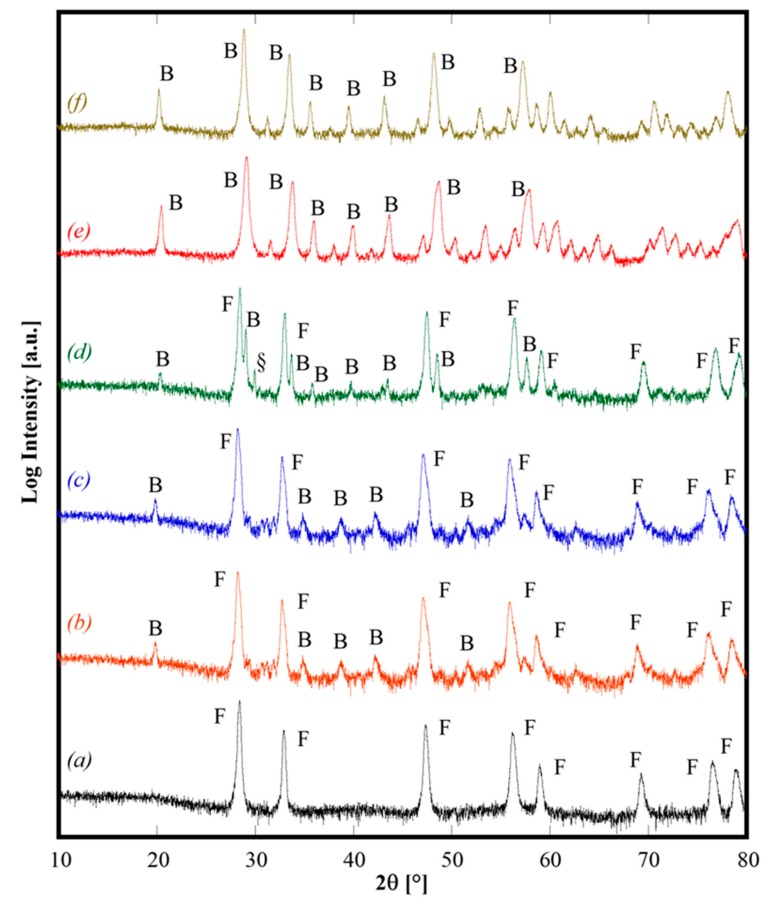
Diffraction patterns of CeZrYLaGd-N (pattern *a*), CeZrYLaGd-U (pattern *b*), CeZrYbLaGd-U (pattern *c*), CeZrYbLaEr-U (pattern *d*), CeZrYYbEr-U (pattern *e*), CeZrYbErGd-U (pattern *f*) annealed at 1500°C for 1 h and air quenched from 1200 °C. F, B and § label fluorite structure, bixbyite structure and the unknown phase, respectively.

Generally speaking, the as-synthesized products deriving from the hydrothermal treatment are partially crystallized, and have complex phase compositions, particularly when urea is used as the precipitating/mineralizing agent. As an example, Figure 1 shows the diffraction patterns of CeZrYLaGd-N, CeZrYLaGd-U, CeZrYbLaGd-N, CeZrYbLaGd-U. The patterns are clearly complex, presenting multiple phases and the as-synthesized samples are only partially crystallized. In the case of samples synthesized with NH_3_ (Figure 1, patterns *a* and *c*), the related patterns are rather similar, exhibiting the presence of a fluorite-like phase labeled as F in Figure 1 (very probably a substitutional solid solution of CeO_2_ and ZrO_2_), and of: either Y (in CeZrYLaGd-N) or Yb (in CeZrYbLaGd-N), Gd and La hydroxides, ICDD cards n. 73-1795 (or 76-1495), 83-2037 and 83-2034, respectively. These four hydroxides, which crystallize in the same space group (n. 176) and have similar ionic radii, exhibit similar Bragg’s angles and as they are indistinguishable in the case of such broad peaks, are collectively labeled as H in Figure 1. On the contrary, when urea was used as a mineralizing agent, its thermal decomposition led to an environment containing carbonate ions, with the result that the as-synthesized phases are mainly based on carbonates. This feature is clearly evident in Figure 1, as the related diffraction patterns (Figure 1, patterns *b* and *d*) are much more complex if compared with patterns *a* and *c*. In the case of CeZrYLaGd-U (Figure 1, pattern *b*), all the main peaks can be assigned to a tengerite-based phase, i.e., a hydrated carbonate with formula Y_2_(CO_3_)_3_∙2H_2_O (ICDD card n. 81-1538), labelled as T, although, very probably, Y^3+^ is partially replaced with larger Gd^3+^ and La^3+^ cations, as indicated by the negative shift of the Bragg’s angles. The formation of the tengerite phase during hydrothermal processes in the presence of carbonate environments was recently reported by the authors for several rare earths, including Y and Gd [23], so this finding is not unexpected. In addition, the evident amorphous halo present at about 30° 2θ is related to hydrous zirconia [17] whereas cerium give rise to cerianite (CeO_2_), whose main peaks are labeled with F in Figure 2, pattern *b*. Finally, in the case of CeZrYbLaGd-U (Figure 1, pattern *d*), the formation of tengerite-phase is hindered by the presence of Yb (replacing Y in this system), thus confirming the results reported in [23]. So, in this extremely complex pattern, it is possible to detect at least four different phases: cerianite, an amorphous phase (related to zirconium compound), kozoite-phase, i.e., La(CO_3_)OH (ICDD card n. 4-009-6412) and ytterbium oxy-carbonate (ICDD card n. 27-955).These findings indirectly confirm that it is not possible to obtain single-phase systems at low temperature with this complex composition, whereas it is well-known that for simpler systems (with two or three different cations), a single-phase of doped and co-doped ceria-based and zirconia-based ceramics, with a fluorite structure, are directly synthesized by hydrothermal treatment in very similar physical-chemical conditions [20,24,25].

All the as-synthesized samples were calcined at 1500 °C for 1 h (by using 10 °C/min as heating rate), followed by air quenching by quickly removing the samples from the furnace when it reached 1200 °C during cooling. The diffraction patterns of some selected calcined samples, shown in Figure 2, demonstrate different behaviors. Sample CeZrYGdLa-N (Figure 2, pattern *a*) is a single phase CeO_2_-like fluorite phase (ICDD card n. 034-0394). Samples CeZrYLaGd-U, CeZrYbLaGd-U and CeZrYbLaEr-U (Figure 2, patterns *b*, *c*, and *d*, respectively) are two-phase systems, one phase is a fluorite crystal structure and the second phase is a bixbyite-like crystal structure (related to Gd_2_O_3_, ICDD card n. 12-797 for the first sample, and related to Er_2_O_3_, ICDD card n. 77-463 for the second one); for the sample CeZrYbLaEr-U, very probably, a minor phase is also present as a non-assigned peak, labeled in Figure 2, pattern *d* with “§”, which appears at about 30° 2θ. Finally, the last two samples, CeZrYYbEr-U (Figure 2, pattern *e*) and CeZrYbGdEr-U (Figure 2, pattern *f*), are single phase bixbyite-like crystal structures in both cases corresponding to ICDD card n. 77-463. These results agree well with those of Sarkar [5], who reported the formation of bixbyite single phase products, by calcination at 1000 °C, for systems with similar composition, even though for three samples, the presence at high temperature of a fluorite-structured phase is revealed, whereas Sarkar obtained the fluorite structure only at low temperature. Very likely, the presence of Zr—a second cation able to promote the fluorite crystal structure—in our samples explains this difference.

Data reported in Figure 2 allow us to derive some interesting considerations. Upon calcination at 1500 °C for 1 h and subsequent quenching, samples with slightly different composition can exhibit really different behaviors (see, for example, Figure 2, patterns *d* and *e*) but even samples owning the same composition, i.e., CeZrYLaGd, exhibit a different behavior if either are synthesized by using urea or ammonia (see Figure 2, patterns *a* and *b*). The former finding could be related to the effect of ionic radii influencing the enthalpic contribution as a consequence of stresses introduced inside the lattice when a substitutional solid solution is formed. Conversely, the latter finding seems related to different reactivities of the powders as a consequence of their different “synthesis history” (i.e., if either ammonia or urea is used as precipitating/mineralizing agent). Considering the different as-synthesized phases formed by hydrothermal treatment for the sample CeZrYLaGd, a logical inference is that hydroxides (or hydrated oxides) of these cations might mix at a small scale and then dissolve, in the solid state, thereby forming a single fluorite phase at high temperature. On the contrary, when the as-synthesized phases are carbonate-based, their thermal decomposition lead to Y, Gd and La oxides owning bixbyite structure, forming mechanical mixtures with Ce, Zr oxides with a fluorite structure. Probably, in this case, the involved oxides are not mixed at a sufficiently small scale. As a consequence of that, these two phases remained separated, at least with our temperature and duration of thermal treatment. In other words, this situation resembles a mixture of different oxides, without sufficient solid-state mixing. Therefore, it is possible that in some of the analyzed samples, at temperatures higher than 1500 °C, the reversible entropy-driven transformation in a single fluorite-like phase could possibly occur. Anyway, an in-depth analysis of these further theoretical aspects was outside the scope of the present work, being under development and implementation in a forthcoming paper. Moreover, following the suggestion of Chen [3], for some of the samples not showing the formation of fluorite single-phase (CeZrYbLaGd-U and CeZrYbErGd-U) additional annealing at lower temperatures (i.e., 1300 °C for 4 h) or prolonged annealing at 1500 °C for 4 h were carried with the aim to reveal if the phases highlighted in Figure 2 were metastable ones. The XRD patterns (not reported here) of these samples did not show any significant change of phase with respect the observations of the patterns in Figure 2. Thus, our attention was devoted to the sample with a single-phase fluorite with the aim to detect if that phase is indeed stabilized by entropy.

**Figure 3:**
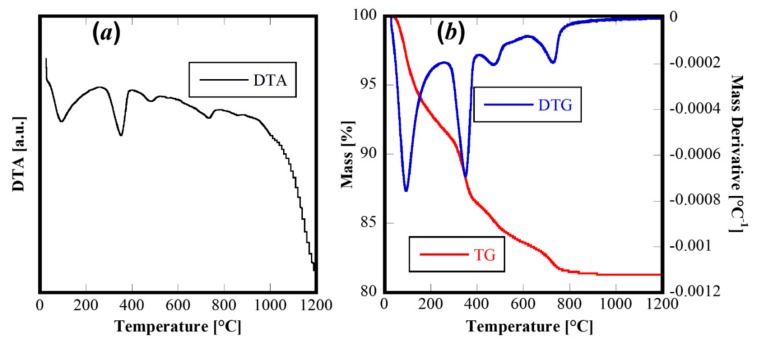
DTA (***a***) and TG/DTG (***b***) of sample CeZrYGdLa-N.

The thermal behavior of this material was revealed by Differential Thermal Analysis and Termogravimetric Analysis (DTA-TG) carried out on the as-synthesized powders to 1200 °C and using 10 °C/min as the heating rate. The corresponding thermograms are displayed in Figure 3. The TG and derivative TG (Figure 3b) reveal a rather complex behavior with four different thermal decomposition events (well highlighted by the derivative of TG curve-DTG curve in Figure 3b). The first one, at about 100 °C, is related to the evolution of adsorbed water on the powders, whereas the others (at about 400 °C, 500 °C and 750 °C, respectively) are related to thermal decomposition of hydroxides or hydrated oxides formed during the hydrothermal treatment. The four evident endothermic peaks in the DTA curve can be associated with the decomposition steps, with no other thermal events detectable. Therefore, the thermal analysis confirms the complex system derived from the hydrothermal treatment and suggested by XRD analysis.

The lattice parameter of CeZrYGdLa-N—obtained by unit cell refinement using the software UnitCell [26]—is 0.54246(1) nm, an intermediate value between the lattice parameter of 20% Gd-doped ceria and the one of 30% Gd-doped ceria. Therefore, the structure of this single-phase equiatomic system is fluorite-ceria, with cations formed by an equimolar mix of Ce, Zr, Y, La, and Gd. In order to balance the charges, a large amount of oxygen vacancies (much more than in any of the doped or non-stoichiometric binary rare earth oxides) is present in this sample because roughly the 60% of the cations composing the system are present in the 3+ oxidation state rather than 4+. Clearly, the presence of a remarkable concentration of oxygen vacancies leads to a significant increase of the configurational entropy [3].

Therefore, the mixed oxide Ce_0.20_Zr_0.20_Y_0.20_Gd_0.20_La_0.20_O_1.70_ could be a potential ESO candidate, because a complete substitutional solid solution for this composition should be excluded on the basis of the factors determining the extended solid solution in oxides. As reported by Kingery [27], the main factors determining the extended solubility in oxides (based on Gibbs free energy variations), are size and valence. However, the cations that compose this sample have two different valencies (4+ for Ce and Zr, and 3+ for Y, La, and Gd) and an adequately different size between the smallest cation (for Zr^4+^ r = 0.084 nm in VIII coordination) and the largest (for La^3+^ r = 0.116 nm in VIII coordination). Of course, other probable explanations are possible, e.g., a non-equilibrium system. In this regard, following the suggestion by Chen [3], to demonstrate that the oxide formed is predominantly stabilized by entropy, it needs to be shown that it can be transformed to a multiphase system by annealing at lower temperatures, and the multiphase state can be reversibly transferred back to the original single-phase system by annealing at higher temperature.

**Figure 4:**
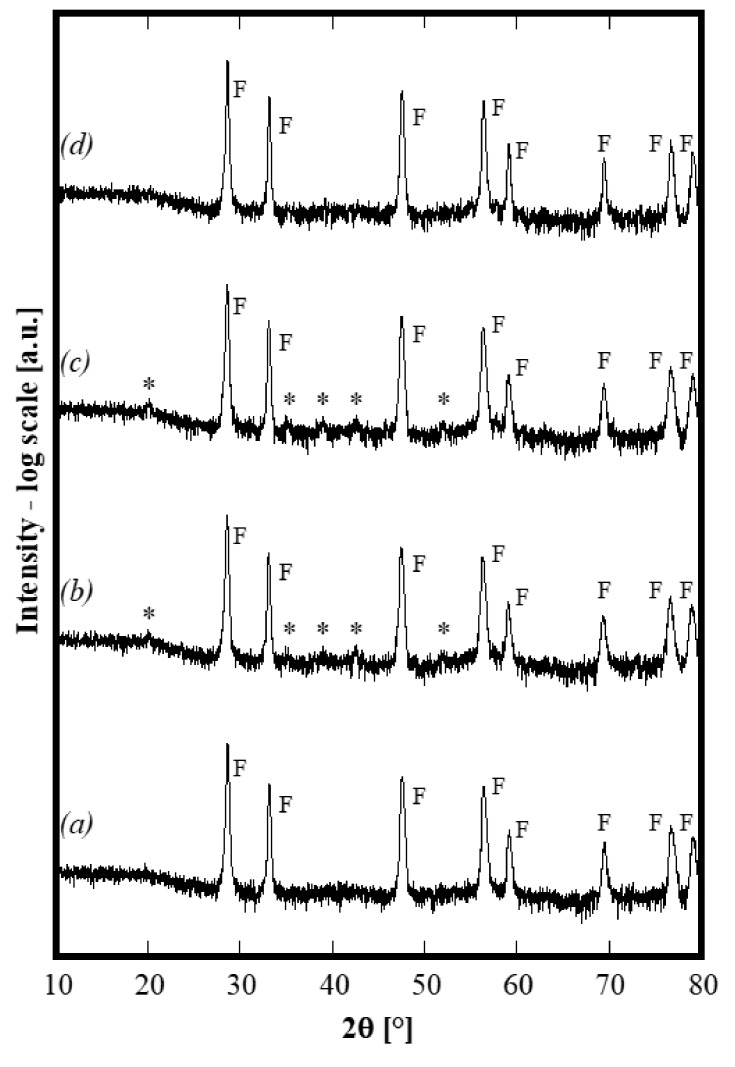
Diffraction patterns of same sample CeZrYGdLa-N first annealed at 1500 °C (pattern ***a***), then 750 °C (pattern***b***), then 1100°C (pattern ***c***) and then again 1500 °C (pattern***d***).

A series of thermal treatments on the same sample, previously treated at 1500 °C, was carried out at selected temperatures in the following order: 750 °C for 4 h, 1100 °C for 4 h and finally, 1500 °C for 4 h. In the first two cases, air quenching was carried out directly from the peak temperature, whereas, in the last case, the quenching was carried out when the furnace was at 1200 °C. At the end of each annealing, XRD spectra were recorded and the corresponding diffraction patterns are displayed in Figure 4 (in which the axis of intensity is in log-scale to highlight the presence of low-intensity peaks). By carefully analyzing such patterns, it is apparent that either after the annealing at 750 °C or after the annealing at 1100 °C, the sample is no longer single phase because of the presence of several small (but not negligible) additional peaks labeled with a star in Figure 4, patterns *b* and *c*. All of these small peaks can be assigned to bixbyite Gd_2_O_3_ (ICDD card n. 12-797). Upon thermal treatment at 750 °C, the exsolution of a secondary phase (gadolinium oxide or perhaps a substitutional solid solution of Y and/or La in gadolinium oxide) occurred, with this secondary phase structure still stable at 1100 °C. As a further confirmation, the lattice parameter calculated for the fluorite-like phase in the sample annealed at 1100 °C is 0.54290(1) nm, i.e., slightly larger than that of the samples calcined at 1500 °C. This result is perfectly compatible with the exsolution of a small amount of Gd_2_O_3_ since it demonstrates that the average cationic radius of trivalent cations increases when the amount of Gd decreases, by consequently making the lattice parameter increase. On the contrary, upon annealing at 1500 °C (corresponding to an air quenching starting from 1200 °C), the system becomes single phase. The lattice parameter of this single phase decreases again to 0.54258(1) nm, i.e., very close to 0.54246 nm. Thus, after completing various annealing cycles, the sample exhibits exactly the same structure obtained by the first thermal treatment at 1500°C. Therefore, the entropy stabilization of this 5-component oxide is clearly established. Furthermore, by considering that the quenching from 1200 °C “freezes” the phases at such a temperature and that at 1100 °C, the system was multiphase, it is possible to theorize that the entropy-driven stabilization occurs at a temperature ranging from 1100 to 1200 °C. To the best of our knowledge, it is the first time that ESO with this composition containing a fluorite structure has been synthesized. However, these preliminary results suggest that succeeding in the stabilization of the single phase, for some systems, could be due to the annealing temperature. In fact, in the case of a very similar composition, i.e., using Yb instead of Y, the system is still a two-phase system after annealing at 1500 °C for 1 h. However, considering the low intensity of the peaks ascribed to the secondary bixbyite phase, its amount is low and a thermal treatment at higher temperature could stabilize the single phase, as a consequence of the higher ΔH of mixing.

**Figure 5:**
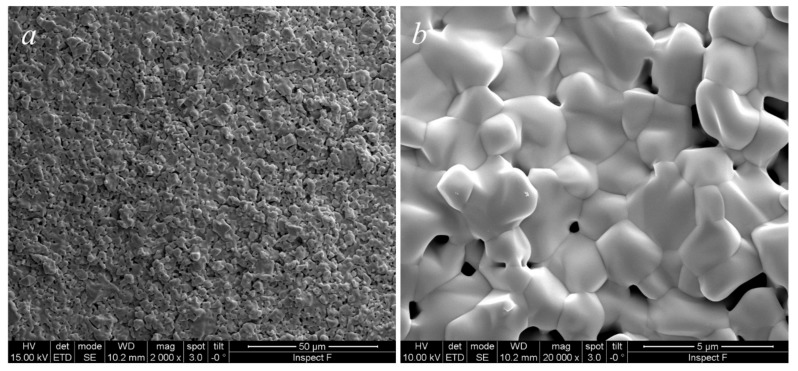
SEM micrographs of CeZrYLaGd-N sintered at 1500°C for 4 h at lower magnification (***a***) and higher magnification (***b***).

These powders were finally formed by uniaxial pressing at 1100 tons and then fired at 1500 °C for 5 h, followed by air-quenching when the furnace was at 1200 °C. The sample is sufficiently densified as can be observed in the SEM micrograph in Figure 5a; the grains structure is quite homogeneous with equiaxed grains whose size is in the order of some µm (Figure 5b). This microstructure strongly suggests the presence of a single phase in the specimen, confirming the results obtained from the annealing step. A porosity homogeneously distributed in the specimens, localized, in general, along the grain boundary, having a size of some µm, is clearly visible. Therefore, based on this microstructure, it is reasonable to exclude the presence of hard agglomerates in the specimen that could be formed during the calcination step. In fact, the microstructure of sintered ceria-based samples prepared from agglomerated powders differs greatly [22]. Most likely, the specimens did not reach full densification because of an incomplete third stage of sintering, maybe related to a reduced reactivity of the powder as a result of the high calcination temperature. Despite that, this result is still rather satisfactory as it is the first sintering attempt of this novel material. A higher degree of densification may be obtained by optimizing both calcination and sintering cycles.

The chemical analysis obtained through EDS, reported in Table 1, shows results that are close to the theoretical composition; in fact, with the exception of Zr, the at% of all other elements is very similar to each other. Considering the qualitative results of the EDS characterization, the data reported in Table 1 are highly compatible with the used nominal composition. The mapping of the various cations confirms their perfectly homogeneous distribution, which is also indicated from the microstructure revealed from SEM micrographs, again indicating the presence of a single phase.

Finally, as said before, it is possible that some other candidates with a composition similar to those studied in this work can exhibit the entropy-stabilization of fluorite-like single-phase, for example, by using a higher temperature of annealing. Therefore, further studies are necessary, with the objective of discovering novel ESOs and elucidating which of the parameters influence the formation of a single-phase ESO rather than a multiphase oxide or a HEO.

## 4. Conclusions

In this work, homogeneous precursors of 5-component (Ce,Zr,Y,La,Gd) ESOs entropy-stabilized in a fluorite single-phase in the temperature range 1100–1200 °C were successfully synthesized starting from powder precursors obtained by hydrothermal treatment at low temperature in the presence of NH_3_ as the precipitating/mineralizing agent. At the same time, samples derived from urea with the same or very similar compositions, did not demonstrate entropy-driven stabilization, at least in the temperature range analyzed in the present study. A possible effect of the as-synthesized phases on the transition to single-phase entropy-stabilized oxides is proposed. Moreover, the obtained powders of (Ce,Zr,Y,La,Gd) oxide exhibit a good sinterability. 

## Figures and Tables

**Figure 6:**
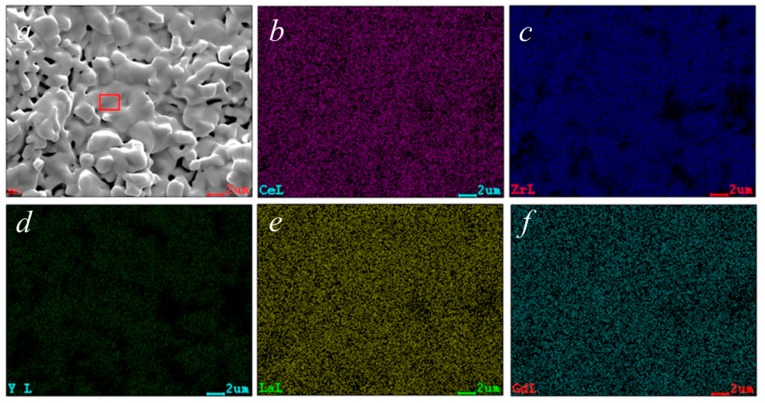
Scanned area for chemical analysis by EDS (Figure 6***a***) and mapping of all cations in the area showed in Figure 6***a***: Ce (***b***), Zr (***c***), Y (***d***), La (***e***) and Gd (***f***).

**Table 1:** Chemical analysis by EDS of area highlighted in Figure 6*a.*

Element	At%
Ce	11.6
Zr	15.9
Y	10.4
La	12.1
Gd	11.7
